# Some *Strychnos spinosa* (Loganiaceae) leaf extracts and fractions have good antimicrobial activities and low cytotoxicities

**DOI:** 10.1186/1472-6882-14-456

**Published:** 2014-11-27

**Authors:** Adamu Imam Isa, Maurice Ducret Awouafack, Jean Paul Dzoyem, Mohammed Aliyu, Rabiu AbduSsalam Magaji, Joseph Olusegun Ayo, Jacobus Nicolaas Eloff

**Affiliations:** Phytomedicine Programme, Department of Paraclinical Sciences, Faculty of Veterinary Science, University of Pretoria, Private Bag X04, Onderstepoort, 0110 South Africa; Department of Human Physiology, Faculty of Medicine, Ahmadu Bello University, Zaria, Nigeria; Laboratory of Natural Products Chemistry, Department of Chemistry, Faculty of Science, University of Dschang, P.O. Box 67, Dschang, Cameroon; Department of Biochemistry, Faculty of Science, University of Dschang, P.O. Box 67, Dschang, Cameroon; Department of Physiology, Faculty of Veterinary Medicine, Ahmadu Bello University, Zaria, 81001 Nigeria

**Keywords:** Extracts, Different polarity fractions, Antimicrobial, Antioxidant, Cytotoxicity, Selectivity index, Potentizing extracts

## Abstract

**Background:**

*Strychnos spinosa* Lam. is a deciduous tree used in traditional medicine to treat infectious diseases. This study is designed to determine the antimicrobial, antioxidant and cytotoxic activities of extracts and fractions from leaves of *S. spinosa.*

**Methods:**

Extracts were obtained by maceration with acetone, methanol and dichloromethane/methanol (1/1) while fractions were prepared by liquid-liquid fractionation of the acetone extract. A broth serial microdilution method with tetrazolium violet as growth indicator was used to determine the minimum inhibitory concentration (MIC) against fungi, Gram-positive and Gram-negative bacteria. The antioxidant activity was determined using free-radical-scavenging assays, and the 3-(4,5-dimethylthiazolyl-2)-2,5-diphenyltetrazolium bromide reduction assay was used to determine cytotoxicity.

**Results:**

Four extracts and five fractions had good to weak antimicrobial activity with MICs ranging from 0.04 to >1.25 mg/ml against both fungi and bacteria. The chloroform and ethyl acetate fractions had an MIC of 0.08 mg/ml against *Aspergillus fumigatus*. The n-butanol fraction had an MIC of 0.04 mg/ml against *Cryptococcus neoformans*. The hexane and chloroform fractions had an MIC of 0.08 mg/ml against *Staphylococcus aureus*. The antioxidant activities were much lower than that of the positive controls. Except for the alkaloid extract, all the extracts and fractions had free-radical-scavenging activity (IC_50_ ranging from 33.66 to 314.30 μg/ml). The cytotoxicity on Vero cells was reasonable to low with LC_50_ values ranging between 30.56 and 689.39 μg/ml.

**Conclusion:**

The acetone extract and the chloroform fraction had the highest antibacterial activity. By solvent-solvent fractionation it was possible to increase the activity against *A. fumigatus* and to decrease the cytotoxicity leading to a potentially useful product to protect animals against aspergillosis. Our results therefore support the use of *S. spinosa* leaves in traditional medicine to treat infectious diseases.

## Background

The genus *Strychnos* is a member of the Loganiaceae family comprising about 200 species. Plant species of this genus have been used in folk medicine and in arrow and dart poisons in many parts of the world [1]. *Strychnos spinosa* Lam. is deciduous shrub or small tree up to 10 m tall, with a trunk sometimes fluted, up to 25 cm in diameter, branching from low down. The fruit is generally considered by botanical collectors to be edible, but there are poison-makers who express a contrary opinion and consider the unripe fruit poisonous [2]. It is widely spread in Africa from Senegal through tropical Africa to South Africa. One of its vernacular name given by Hausa people in Nigeria is “*Kwokwa* or *Kokiya”*[2]. The plant species is used in traditional medicine for treating snakebite, ulcers, wounds, headache, gastric and intestinal problems, venereal diseases, leprosy, diarrhea, and fever [2]. In Gambia, the plant’s leaf decoction with barks powder are used for the treatment of wounds while in Cameroon dried powdered leaves are taken in food for liver damage [2]. The Zulu of South Africa use the green fruits as an antidote to snakebite [3, 4]. Many pharmacological properties including antiplasmodial [5] antitrypanosomal [6, 7] and anthelmintic [8] activities have been reported from *S. spinosa*. Extracts of the stem bark of *S. spinosa* had no activity against bacteria or fungi [9]. The antioxidant activity of the fruit extract of the same species was determined [10]. Several secondary metabolites including triterpenoids, sterols and essential oils [6, 7] secoiridoids [11, 12], alkaloids [13], and monoterpenes [14] have been indicated or isolated from *Strychnos spinosa*.

Despite the fact that the leaves of the plant are used in folk medicine in the treatment of several infectious diseases, there is paucity of scientific evidence of the antimicrobial and antioxidant activities, and cytotoxicity of its leaf extract. The aim of this presentation was to determine the antimicrobial and antioxidant activities and also the cytotoxicity of four extracts and five fractions obtained by liquid-liquid extraction from leaves of *S. spinosa*.

## Methods

### Plant material

The leaves of *Strychnos spinosa* Lam. were collected in January 2013 from Sakara village, Zaria, Nigeria. The plant material was identified by Musa Muhammad a botanist from the herbarium section of the Department of Biological Sciences (Ahmadu Bello University, Zaria) where a voucher specimen (No 900161) was deposited. The collected leaves were dried in a ventilated room free from contamination and then ground to a powder using a Macsalab Mill (Model 2000 LAB Eriez), kept in a glass container and stored in the dark at room temperature (25 ± 3°C) before use.

### Extraction and liquid-liquid fractionation

#### Acetone, methanol and dichloromethane/methanol extractions

A variation of the method of Suffness & Douros [15] was used to fractionate the components present in different leaf extracts. The dried leaf powder (2 kg) was macerated three times in acetone (6 l) [16] to give the acetone extract (AcetE, 75 g) after filtration and removal of the solvent in vacuum. The residues were further macerated in methanol (6 l) following the same procedure as described for acetone extraction above to afford the methanol extract (MetE, 119.2 g). A part of the dried powdered leaves (1 kg) was also extracted in a mixture (1/1, v/v) of dichloromethane/methanol (3 l) thrice to give the dichloromethane/methanol extract (DcmMetE, 114 g) after filtration and removal of the solvent in vacuum. A part of acetone extract (70 g) was dissolved and fractionated in a mixture (1/1, v/v) of chloroform and water to yield the water and chloroform fractions. *n*-butanol was added to the water fraction to afford the *n*-butanol (nButF, 25.1 g) and water (Wat1, 5 g) fractions. The chloroform fraction was concentrated to dryness and dissolved in 10% water in methanol before the extraction with hexane. The hexane fraction (HexF, 23. 9 g) and the residue of 10% water in methanol were therefore obtained after addition of n-hexane. The proportion of water in methanol was increased to afford 35% water in methanol component that finally gave chloroform (ChlF, 7.05 g) and 35% water (wat2, 2.8 g) fractions after addition of chloroform. From the comparative TLC, water (Wat1) and 35% water in methanol (Wat2) fractions were combined into one fraction (WatF, 7.8 g).

### Alkaloids extraction

The leaves of *S. spinosa* (1 kg) were macerated with the mixture (96:3:1, v/v) of EtOAc-EtOH-NH_4_OH (600 ml) and then percolated with EtOAc to give the extract (26 g) after removal of the solvent using rotary evaporator under reduced pressure. The extract was dissolved in EtOAc and extracted with 4% acetic acid to afford EtOAc fraction (EtAcF, 20.02 g). The acidic solution (pH 3–4) was basified to pH (8–9) with Na_2_CO_3_ and extracted three times with DCM to give crude alkaloids extract (AlkE, 2.8 g) after removal of the solvent in vacuum.

### Antimicrobial assay

#### Microorganisms and inoculum preparation

Microorganisms used were three Gram-positive bacteria, *Bacillius cereus* (ATCC 14579), *Staphylococcus aureus* (ATCC 29213) and *Enterococcus faecalis* (ATCC 29212), one Gram-negative bacteria, *Escherichia coli* (ATCC 25922); and four fungi including three yeast *Candida albicans*, *Cryptococcus neoformans* (animal isolates) and *Candida albicans* (ATCC 10231) and one filamentous fungi *Aspergillus fumigatus*. Some fungal strains used were cultured from clinical cases of fungal infectious diseases in animals, before treatment, in the Department of Veterinary Tropical Diseases, Faculty of Veterinary Science. *C. albicans* was isolated from a Gouldian finch, *C. neoformans* from a cheetah, while *A. fumigatus* was isolated from a chicken which suffered from a systemic mycosis.

Bacterial and fungal cultures were taken from 24 h fresh agar culture plates and inoculated in fresh Sabouraud dextrose broth (SDB) for fungi and Mueller-Hinton broth (MHB) (Fluka, Switzerland) for bacteria, prior to conducting the assay. The turbidity of the microbial suspension was adjusted to a McFarland standard 0.5 equivalent to concentrations of 1–5 × 10^8^ and 1–5 × 10^7^ cfu/ml for bacteria and fungi, respectively. The microbial suspensions were further diluted (1:100) in media to obtain a final inoculum of approximately 1.5 × 10^6^ cfu/ml for bacteria and 1.5 × 10^5^ cfu/ml for fungi.

### Minimun inhibitory concentration determination

A two-fold serial microdilution method with tetrazolium violet as indicator of microbial growth was used to determine the minimum inhibitory concentration (MIC) values for extracts and fractions against bacteria [16] and fungi [17] as modified by Masoko et al. [18].

A 100 μl (10 mg/ml) of extracts and fractions dissolved in dimethylsulfoxide (DMSO) were serially diluted two-fold with sterile distilled water in 96-well microtitre plates and 100 μl of freshly prepared microbial culture in MHB or SDB was added to each well. DMSO (5%) was used as negative control while (1 mg/ml) gentamicin and amphotericin B were positive controls. The microtitre plates were sealed in plastic bags and incubated for 24 h at 37°C. Thereafter, 40 μl of 0.2 mg/ml of *p*-iodonitrotetrazolium violet (INT) was added to each well and microtitre plates were further incubated at 37°C. Minimal inhibitory concentrations were determined after 1 and 2 h for bacteria, and 16 and 36 h for fungi. The MIC was determined as the lowest concentration inhibiting microbial growth, indicated by a decrease in the intensity of the red color of the formazan.

### Antioxidant assays

The antioxidant activities of extracts and fractions from the leaves of *S. spinosa* were determined in term of free-radical scavenging ability using 2,2′-diphenyl-1-picryhydrazyl (DPPH) and 2,2-azino-bis (3-ethylbenzothiazoline-6-sulphonic acid) diammonium salt (ABTS).

### DPPH assay

The antioxidant activity was performed as described by Du Toit et al. [19] with slight modifications. Samples were dissolved in HPLC-grade methanol (Sigma-Aldrich, Germany) and two-fold serially diluted to concentration ranges of 1000 to 7.81 μg/ml for extracts and fractions, and 40 to 0.31 μg/ml for a standard reference L-ascorbic acid (Sigma, Germany). Briefly, 40 μl of (10 mg/ml) of samples were introduced in a 96-well microtitre plates (Bioster, Spain) and two-fold serially diluted in methanol. Thereafter, 160 μl of (3.7 mg/100 ml) methanolic solution of 2,2-diphenyl-1-picryhydrazyl (DPPH) was introduced in each well and after 30 min incubation at room temperature in the darkness and the absorbance was measured at 517 nm using a Multi-Mode Microplate Reader (BioTek, USA). The free-radical-scavenging activity of each sample and the reference standard were determined as percent of the inhibition obtained from the following formula:


With Ab_sample_ as the absorbance of the extract with DPPH, Ab_blank_ as the absorbance of the extract without DPPH and Ab_control_ as absorbance of methanol and DPPH. The concentration of samples reducing 50% of free-radical DPPH (IC_50_) was determined by plotting the percentage of inhibition against the sample concentrations. The assay was replicated three times and results are expressed as mean ± standard deviation.

### ABTS assay

The ABTS radical-scavenging capacity of extracts and fractions was determined using a method of Re et al. [20] with slight modifications. Briefly, the ABTS radical was generated by reacting 7 mM solution of ABTS and 2.45 mM solution of potassium persulfate at room temperature for 12 h. The absorbance of ABTS radical stock solution was adjusted to 7.00 ± 0.02 at 734 nm before used. 40 μl of a solution of extracts or fractions dissolved in HPLC-grade methanol (Sigma-Aldrich, Germany) were introduced to microtitre plates and tow-fold serially diluted to concentrations range of 15.62 and 2000 μg/ml. Trolox (Sigma, Germany) and L-ascorbic acid (Sigma, Germany) were prepared in concentrations ranging from 200 to 1.56 μg/ml. Thereafter, 160 μl of ABTS solution was added to wells (except the blank) and the absorbance was measured at 734 nm after 6 min incubation at room temperature. Trolox and ascorbic acid were used as positive controls, methanol as negative control and extracts or fractions without ABTS as blank. Percentage of ABTS•+ inhibition and IC_50_ were calculated as reported above for DPPH assay.

### Cytotoxicity assay

The cytotoxicity of the acetone extracts and fractions against Vero monkey kidney cells was assessed by the MTT reduction assay as previously described [21] with slight modifications. Cells were seeded at a density of 1 × 10^5^ cells/ml (100 μl) in 96-well microtitre plates and incubated at 37°C and 5% CO_2_ in a humidified environment. After 24 h incubation, samples (100 μl) at varying final concentrations were added to the wells containing cells. Doxorubicin was used as a positive reference. A suitable blank control with equivalent concentrations of acetone was also included and the plates were further incubated for 48 h in a CO_2_ incubator. Thereafter, the medium in each well was aspirated from the cells, which were then washed with PBS, and finally fresh medium (200 μl) was added to each well. Then, 30 μl of MTT (5 mg/ml in PBS) was added to each well and the plates were incubated at 37°C for 4 h. The medium was aspirated from the wells and DMSO was added to solubilize the formed formazan crystals. The absorbance was measured on a BioTek Synergy microplate reader at 570 nm. Cell growth inhibition for each extract was expressed in terms of LC_50_ values, defined as the concentration that caused 50% of inhibition of cell viability. The selectivity index (SI) values were calculated by dividing cytotoxicity LC_50_ values by the MIC values (SI = LC_50_/MIC). Tests were carried out in quadruplicate and each experiment was repeated thrice.

### Statistical analysis

All experiments were conducted in triplicate and values expressed as mean ± standard deviation. Differences between values were assessed for significance using analysis of variance and results were compared using the Fisher’s least significant difference (LSD) at 5% significance level.

## Results and discussion

### Extraction yield

Dried powdered leaves of *Strychnos spinosa* were extracted with acetone, methanol, mixture (v/v) of dichloromethane/methanol (1/1) and alkaloids extraction procedure to afford extracts (AcetE, MetE, DcmMetE and AlkE) with yields of 3.7, 11.9, 11.0, and 0.28%, respectively.

### Antimicrobial activities

Antimicrobial of leaf extracts and fractions of *S. spinosa* were determined against four fungi and four bacteria and the results are given as minimum inhibitory concentrations (MIC) and total activity in Tables 1 and 2. Many authors classified the antimicrobial activity of plant extracts and fractions to be significant if the MIC is 0.1 mg/ml or lower, moderate if 0.1 < MIC ≤0.625 mg/ml and weak if MIC >0.625 mg/ml [22, 23]. Based on these criteria, the test samples had significant to weak antimicrobial activity with MICs ranging from 0.04 to >1.25 mg/ml against both fungi and bacteria (Table 1).

**Table 1:** **Minimun inhibitory concentration (MIC in mg/ml) of extracts and fractions from** ***S. spinosa*** **against fungi and bacteria**

	Fungi ^a^								Bacteria ^b^							
Samples	C. a		C.A		A.f		C.n ^c^		S.a		B.c		E.f		E.c	
	16 h	24 h	16 h	24 h	16 h	24 h	24 h	36 h	1 h	2 h	1 h	2 h	1 h	2 h	1 h	2 h
**Extracts** ^**d**^																
AcetE	>1.25	>1.25	>1.25	>1.25	1.25	>1.25	0.63	0.63	0.16	0.16	0.32	0.32	0.16	0.16	0.32	1.25
MetE	>1.25	>1.25	>1.25	>1.25	1.25	1.25	0.63	0.63	>1.25	>1.25	>1.25	>1.25	>1.25	>1.25	0.63	0.63
DcmMetE	>1.25	>1.25	>1.25	>1.25	1.25	1.25	0.16	0.16	0.63	0.63	>1.25	>1.25	0.63	0.63	0.63	0.63
AlkE	>1.25	>1.25	>1.25	>1.25	>1.25	>1.25	0.32	0.32	0.16	0.16	1.25	>1.25	0.32	0.32	0.16	1.25
**Fractions** ^**e**^																
HexF	1.25	1.25	0.63	0.63	>1.25	>1.25	0.32	1.25	**0.08**	**0.08**	0.63	1.25	0.32	0.32	**0.08**	1.25
ChlF	0.16	0.16	0.63	0.63	**0.08**	**0.08**	0.32	1.25	**0.08**	**0.08**	0.63	0.63	0.16	0.16	0.16	0.32
EtAcF	0.63	0.63	0.16	0.16	**0.08**	**0.08**	0.32	1.25	0.16	0.16	0.63	1.25	0.16	0.32	0.16	1.25
nBuF	>1.25	>1.25	>1.25	>1.25	1.25	1.25	**0.04**	1.25	**0.08**	0.16	0.63	0.63	1.25	1.25	0.63	0.63
WatF	>1.25	>1.25	>1.25	>1.25	1.25	1.25	0.63	>1.25	>1.25	>1.25	>1.25	>1.25	>1.25	>1.25	1.25	>1.25
**Controls** ^**f**^																
Amp B	16	16	8	8	16	16	> 250	> 250	-	-	-	-	-	-	-	-
Gen	-	-	-	-	-	-	-	-	0.78	0.78	0.39	0.39	1.56	1.56	0.39	0.39

^**a)**^C.a: *Candida albicans*(Isolate); C.A: *Candida albicans* (ATCC strain*)*; C.n: *Cryptococcus neoformans;* A.f: *Aspergillus fumigatus,* ^**b)**^E.c: *Escherichia coli*; E.f: *Enterococcus faecalis*; S.a: *Staphylococcus aureus*; B.c: *Bacillus cereus,* ^**c)**^with this microorganism litle reaction was observed after 16 h and MIC were recorded after 24 h and 36 h, ^**d)**^AcetE: Acetone Extract, MetE: Methanol extract, DcmMetE: Dichloromethane/methanol extract, AlkE: Alkaloids extract, ^**e)**^HexF: n-hexane fraction, ChlF: Chloroform fraction, EtAcF: Ethyl acetate fraction, nBuF: n-Butanol fraction, WatF: Water fraction, ^**f)**^Amp B: Amphotericin B (in μg/ml), Gen: *Gentamicin* (in μg/ml). In bold are values with significant activity.

**Table 2:** **Total activity in ml/g of extracts and fractions from** ***S. spinosa*** **against fungi and bacteria**

	Fungi ^a^								Bacteria ^b^							
Samples	C. a		C.A		A.f		C.n ^c^		S.a		B.c		E.f		E.c	
	16 h	24 h	16 h	24 h	16 h	24 h	24 h	36 h	1 h	2 h	1 h	2 h	1 h	2 h	1 h	2 h
**Extracts** ^**d**^																
AcetE	30	30	30	30	30	30	60	60	**234**	**234**	**117**	**117**	**234**	**234**	**117**	**30**
MetE	48	48	48	48	48	48	95	95	48	48	48	48	48	48	95	95
DcmMetE	46	46	46	46	46	46	**356**	**356**	**90**	**90**	**46**	**46**	**90**	**90**	**90**	**90**
AlkE	2	2	2	2	2	2	9	9	18	18	2	2	9	9	18	2
**Fractions** ^**e**^																
HexF	10	10	19	19	10	10	37	10	**149**	**149**	19	10	37	37	**149**	10
ChlF	23	23	6	6	47	47	12	3	47	47	6	6	23	23	23	12
EtAcF	32	32	**125**	**125**	**250**	**250**	63	16	**125**	**125**	32	16	**125**	63	**125**	16
nBuF	10	10	10	10	10	10	**314**	10	**157**	**78**	20	20	10	10	20	20
WatF	3	3	3	3	3	3	6	3	3	3	3	3	3	3	3	3

^**a)**^C.a: *Candida albicans*(Isolate); C.A: *Candida albicans* (ATCC strain*)*; C.n: *Cryptococcus neoformans;* A.f: *Aspergillus fumigatus,* ^**b)**^E.c: *Escherichia coli*; E.f: *Enterococcus faecalis*; S.a: *Staphylococcus aureus*; B.c: *Bacillus cereus,* ^**c)**^with this microorganism litle reaction was observed after 16 h and MIC were recorded after 24 h and 36 h, ^**d)**^AcetE: Acetone Extract, MetE: Methanol extract, DcmMetE: Dichloromethane/methanol extract, AlkE: Alkaloids extract, ^**e)**^HexF: n-hexane fraction, ChlF: Chloroform fraction, EtAcF: Ethyl acetate fraction, nBuF: n-Butanol fraction, WatF: Water fraction. In bold are values with significant activity.

All the extracts had moderate activity against *C. neoformans* with MICs ranging from 0.16 to 0.63 mg/ml. The other fungi were more resistant to extracts with MICs 1.25 or >1.25 mg/ml. The chloroform and ethyl acetate fractions had significant activities against *A. fumigatus* with MIC 0.08 mg/ml in both cases and were reasonably active against the two strains of *Candida* (MICs 0.16 and 0.63 mg/ml). The n-butanol fraction had good activity against *C. neoformans* (MIC 0.04 mg/ml) after 24 h of incubation and weak activity against other fungi (MICs 1.25 or >1.25 mg/ml). The hexane fraction had moderate activity against *C. albicans* ATCC strains (MIC 0.63 mg/ml) and weak activity against *C. albicans* isolate (MIC 1.25 mg/ml) and *A. fumigatus* (MIC >1.25 mg/ml). Apart from *C. neoformans* (MIC 0.63 mg/ml after 24 h of incubation), all the fungi were relatively more resistant to the water fraction with MICs 1.25 or >1.25 mg/ml.

Most extracts had moderate antibacterial activity with MICs ranging from 0.16 to 0.63 mg/ml. Apart from *E. coli* with MIC value of 1.25 mg/ml after 2 h of incubation, the acetone extract had moderate activity against *S. aureus* (MIC 0.16 mg/ml), *B. cereus* (0.32 mg/ml), *E. faecalis* (MIC 0.16), and *E. coli* (MIC 0.32 mg/ml after 1 h of incubation). The alkaloid extract had moderate activity against *S. aureus* and *E. faecalis* with MICs of 0.16 and 0.32 mg/ml, respectively. Apart from *B. cereus* (MIC >1.25 mg/ml), the dichloromethane/methanol extract had moderate activity against all the bacteria with MICs 0.63 mg/ml in all cases. The hexane and chloroform fractions had significant antibacterial activity against *S. aureus* with an MIC of 0.08 mg/ml in both cases and moderate activity against most of bacteria (MICs 0.16 – 0.63 mg/ml). Good activity was obtained after 1 h of incubation (MIC 0.08 mg/ml) with the n-butanol fraction against *S. aureus* and the hexane fraction against *E. coli*. The water fraction had weak antibacterial activity against all the bacteria (MICs 1.25 mg/ml or greater).

The acetone extract had the highest antibacterial activity (average MIC 0.36 mg/ml) while fungi were resistant to all extracts (average MICs 1.09 mg/ml or greater). These results are similar to those found by previous authors [24, 25]. The chloroform fraction had the highest antibacterial activity (average MIC 0.28 mg/ml) amongst all the fractions, followed by the hexane and ethyl acetate fractions (average MICs 0.50 and 0.51 mg/ml, respectively). These results confirm a statement that the intermediate polarity compounds usually have the highest antimicrobial activity found with many different plant species [26]. The chloroform and the ethyl acetate fractions had most antifungal activity with average MIC of 0.29 mg/ml in both cases, followed by the hexane fraction with average MIC of 0.94 mg/ml.

All the fractions had fungistatic effect against *C. neoformans* while most of the extracts were bactericidal based on the difference in MIC after different time of incubation. Some bacteriostatic effects were observed for acetone and alkaloid extracts against *E. coli*, and some fractions such as n-butanol against *S. aureus*, hexane against *B. cereus* and *E. coli*, chloroform against *E. coli*, and ethyl acetate against *B. cereus*, *E. faecalis* and *E. coli*.

The total activity was obtained to quantify the antimicrobial activity by dividing the mass of extract or fraction from 1 g of the plant material with the MIC value [22]. The acetone extract had the highest antibacterial activity with an average total activity of 165 ml/g. The ethyl acetate fraction had highest antifungal activity with average total activity of 125.30 ml/g on fungi. The dichloromethane/methanol extract had a total activity of 356 ml/g against *C. neoformans*, this implies that 1 g of this extract can be diluted to 356 ml and still inhibits the growth of *C. neoformans*[23, 22]. The MeOH extract from the stem bark of *S. spinosa* was previously investigated and had no antimicrobial activity against *C. albicans*, *S. aureus*, *B. cereus* and *E. coli*[9], this can be explained the fact that acetone dissolves many hydrophilic and lipophylic components from the plants and furthermore substantiate the acetone as the best extractant to be used for the screening of antimicrobial from plant extracts [16].

### Antioxidant activities

Antioxidant activities of different extracts and fractions from the leaves of *S. spinosa* were determined using free-radical-scavenging DPPH and ABTS and results are presented in Table 3. In all cases the antioxidant activity was much lower than that of the positive controls. Apart from the alkaloid extract, all the test samples had DPPH and ABTS-radical-scavenging activity with IC_50_ ranging from 33.66 to 230.15 μg/ml, and 62.74 to 314.30 μg/ml, respectively. The most actives being the water fraction (IC_50_ 33.66 and 65.02 μg/ml), methanol extract (IC_50_ 36.56, 62.74 μg/ml), and *n*-butanol fraction (IC_50_ 42.07, 74.23 μg/ml). Hexane, chloroform and ethyl acetate fractions had the lowest antioxidant activity with IC_50_ ranging from 117.77 to 314.30 μg/ml. Even though, the activity displayed by all the samples was significantly weak compare to ascorbic acid and trolox (p < 0.05). However, the antioxidant activity of the MeOH fruits extract of *S. spinosa* was reported and the free-radical depletion was attributed not only to phenolic contents but also to the presence of traces of vitamin C in the extract [10].

**Table 3:** **Antioxidant activities of extracts and fractions from** ***S. spinosa***

Samples	IC _50_ (μg/ml)	
	DPPH	ABTS
**Extracts** ^**a**^		
AcetE	95.42 ± 0.04^a^	112.20 ± 0.01^a^
MetE	36.56 ± 0.02^b^	62.74 ± 0.01^b^
DcmMetE	59.13 ± 0.02^c^	150.41 ± 0.02^c^
AlkE	-^c)^	-
**Fractions** ^**b**^		
HexF	203.78 ± 0.03^d^	314.30 ± 0.04^d^
ChlF	230.15 ± 0.04^e^	249.82 ± 0.02^e^
EtAcF	117.77 ± 0.04^f^	249.33 ± 0.03^e^
nBuF	42.07 ± 0.03^g^	74.23 ± 0.03^f^
WatF	33.66 ± 0.04^h^	65.02 ± 0.03^g^
**Controls**		
L-Ascorbic acid	4.65 ± 0.02^i^	2.26 ± 0.04^h^
Trolox	9.71 ± 0.04^j^	16.46 ± 0.02^i^

^**a)**^AcetE: Acetone extract, MetE: Methanol extract, DcmMetE: Dichloromethane/methanol extract, AlkE: Alkaloids extract, ^**b)**^HexF: n-hexane fraction, ChlF: Chloroform fraction, EtAcF: Ethyl acetate fraction, nBuF: n-Butanol fraction, WatF: Water fraction, ^c)^no concen- trations decreasing 50% DPPH or ABTS. Values with different letters are significantly different at p < 0.05.

### Cytotoxicity

The cytotoxicity of extracts and fractions was determined on monkey kidney Vero cells *in vitro* by means of the MTT (3-(4,5-dimethylythiazol-2-yl)-2,5-diphenyl-2H-tetrazolium hydrobromide) assay and results are reported in Table 4 together with selectivity indices. All the test samples had LC_50_ values ranging between 30.56 and 689.39 μg/ml. The water fraction was the least toxic extract or fraction followed by ethyl acetate fraction, methanol extract and alkaloid extract with LC_50_ values of 689.39, 479.44, 361.48, 141.54 μg/ml, respectively. The acetone and dichloromethane/methanol extracts had lowest LC_50_ values (30.56 and 38.38 μg/ml) but theirtoxicity was significantly low (p < 0.05) compared to the reference standard doxorubicin (LC_50_ 2.59 μg/ml).

**Table 4:** **Cytotoxicity of extracts and fractions from** ***S. spinosa*** **, and their selectivity index (SI)**

Samples	Cytotoxicity (LC50, μg/ml)	Selectivity index (SI) ^*^							
		C.a	C.A	A.f	C.n	S.a	B.c	E.f	E.c
**Extracts**									
AcetE	30.56 ± 0.00^a^	< 0.02	< 0.02	0.02	0.05	0.19	0.1	0.19	0.04^**^
MetE	361.48 ± 0.02^b^	< 0.30	< 0.30	0.3	0.57	< 0.30	< 0.30	< 0.30	0.57
DcmMetE	38.38 ± 0.00^c^	< 0.03	< 0.03	0.03	0.24	0.06	< 0.03	0.06	0.06
AlkE	141. 54 ± 0.00^d^	< 0.11	< 0.11	< 0.11	0.44	0.88	0.11	0.44	0.20^**^
**Fractions**									
HexF	71.28 ± 0.01^e^	0.06	0.11	< 0.06	0.09^**^	0.9	0.08^**^	0.22	0.11^**^
ChlF	67.20 ± 0.00^f^	0.42	0.11	0.84	0.09^**^	0.84	0.11	0.42	0.28^**^
EtAcF	479.44 ± 0.07^g^	0.76	3	6	0.61^**^	0.76	0.51^**^	0.20^**^	0.68^**^
nBuF	50.49 ± 0.01^h^	< 0.04	< 0.04	0.04	0.08^**^	0.42^**^	0.08	0.04	0.08
WatF	689.39 ± 0.00^i^	< 0.55	< 0.55	0.55	0.73^**^	< 0.55	< 0.55	< 0.55	0.55
**Control**									
Dox	2.59 ± 0.00								

AcetE: Acetone Extract, MetE: Methanol extract DcmMetE: Dichloromethane/methanol extract, AlkE: Alkaloids extract, HexF: n-hexane fraction, ChlF: Chloroform fraction, EtAcF: Ethyl acetate fraction, nBuF: n-Butanol fraction, WatF: Water fraction, Dox: Doxorubicin with LC_50_ in μM, NT: not tested, ^*^SI = LC_50_ /MIC, ^**^SI obtained from average MIC. ) C.^a^ *Candida albicans* (Isolate); C.A: *Candida albicans* (ATCC strain); C.n: *Cryptococcus neoformans*; A.f: Aspergillus fumigatus E.c: *Escherichia coli*, E.f: *Enterococcus faecalis*, S.a: *Staphylococcus aureus*, B.c: *Bacillus cereus*. Values with different letters are significantly different at p < 0.05.

The selectivity index of each extract or fraction on each microorganism was calculated bydividing the LC_50_ by MIC value. It is generally considered that the ratio for a good therapeutic index for a remedy or drug should be >10, which is a cut-off point ensuring that overdose does not put the life of the patient in danger [27]. The acetone extract had the lowest selectivity index against *Candida* (<0.02). The cytotoxicity of the MeOH extract from seeds of *S. nuxvomica* was reported with IC_50_ value of 18134 μg/ ml on MCF-7 cancer cell line while no IC_50_ value was found on Vero cell [28]. The highest selectivity index was obtained for the ethyl acetate fraction against *A. fumigatus* (6.00). These results show that by manipulation of extracts the toxicity can be decreased and/or the efficacy can be increased. It appears that the ethyl acetate fraction has the potential to be used to combat aspergillosis in poultry as was found for an extract of *Loxostylus alata*[29].

## Conclusion

The results obtained support the use of *S. spinosa* in traditional medicine for the treatment of infectious diseases. We are currently busy in isolating and characterizing the constituents responsible for the antimicrobial activity from the most active fractions.
